# GSK3β mediates pancreatic cancer cell invasion in vitro via the CXCR4/MMP-2 Pathway

**DOI:** 10.1186/s12935-015-0216-y

**Published:** 2015-07-05

**Authors:** Xu Ying, Li Jing, Shijie Ma, Qianjun Li, Xiaoling Luo, Zhenguo Pan, Yanling Feng, Pan Feng

**Affiliations:** Department of Gastroenterology, Huai’an First People’s Hospital, Nanjing Medical University, 6 Beijing Road West, Huai’an, Jiangsu 223300 People’s Republic of China; Department of Hepatology, Huai’an Fourth People’s Hospital, No.128, Yan an East Road, Qing pu District, Huai’an, Jiangsu 223300 People’s Republic of China

**Keywords:** Glycogen synthase kinase-3β (GSK3β), CXCR4, MMP-2

## Abstract

**Background:**

Glycogen synthase kinase-3β (GSK3β) expression and activity are upregulated in pancreatic cancer tissues. In our previous study, we found that stromal cell-derived factor-1/ chemokine receptor C-X-C motif chemokine receptor 4 (SDF-1α/CXCR4) upregulated matrix metalloproteinase 2 (MMP-2) and promoted invasion in PANC1 and SW-1990 pancreatic cancer cells by activating p38 mitogen-activated protein kinase (p38 MAPK). Additionally, inhibition of GSK3β reduced MMP-2 secretion.

**Methods:**

To investigate the molecular mechanism of GSK3β in pancreatic cancer tissues, we created stable PANC1 cells up-regulation of GSK3β by transfecting GSK3β overexpression plasmid, and down-regulation of GSK3β using two different types of RNA interference.

**Results:**

Western blotting showed that overexpression of GSK3β up-regulated CXCR4 and MMP-2 expression; suppression of GSK3β down-regulated CXCR4 and MMP-2 protein expression. Up-regulation of MMP2 induced by overexpression of GSK3β was blocked by inhibition of CXCR4. Overexpression of GSK3β promoted PANC1 cell invasion, and down-regulation of GSK3β suppressed PANC1 cell invasion in the transwell invasion assays. However, inhibition of CXCR4 using shRNA attenuated the ability of GSK3β to promote PANC1 cell invasion.

**Conclusions:**

This study demonstrated that GSK3β promotes PANC1 cell invasion via the CXCR4/MMP-2 pathway.

**Electronic supplementary material:**

The online version of this article (doi:10.1186/s12935-015-0216-y) contains supplementary material, which is available to authorized users.

## Background

Pancreatic cancer is one of the most aggressive human cancers worldwide. Despite the introduction of improved and/or combined treatments such as surgery, chemotherapy and radiation therapy, the prognosis of patients with pancreatic cancer remains poor. Novel, effective therapeutic strategies are urgently required to improve the prognosis of patients with this malignancy. Improvements to chemotherapy strategies require the development of novel target-directed therapies. Therefore, further studies are needed to identify novel molecular therapeutic targets which determine the sensitivity of pancreatic cells to chemotherapeutic agents and ionizing radiation [1].

The serine/threonine kinase glycogen synthase kinase-3 (GSK3) was originally identified as a regulator of glycogen synthesis [2, 3]. GSK3β phosphorylates and inactivates the enzyme glycogen synthase. However, GSK3β is also involved in diverse cellular processes such as cell proliferation, apoptosis, invasion and inflammation. Altered GSK3β expression is associated with numerous pathological processes, including type 2 diabetes, Alzheimer’s disease, and cancer [4–7]. Recent studies suggested that GSK3β can promote the invasion of pancreatic, lung, breast and liver cancer cells, and inhibition of GSK3β induces apoptosis [8–10]. These results have led researchers to propose GSK3β as a potential therapeutic target in cancer. Recent studies have also indicated that GSK3β promotes the proliferation and invasion of pancreatic cancer cells. Inhibition of GSK3β triggered an apoptotic response in pancreatic cancer cells via a JNK-dependent mechanism [11], and attenuated cell survival and proliferation and induced apoptosis in pancreatic cancer cell lines [5].

Chemokine receptor C-X-C motif chemokine receptor 4 (CXCR4) and its natural ligand stromal cell-derived factor-1 (CXCL12, SDF-1) play a role in a variety of physiological and pathological processes, including cell proliferation, migration and invasion [12]. Matrix metalloproteinase 2 (MMP-2) promotes the invasion of a variety of cancer cells, including pancreatic cancer cells [13]. SDF-1/CXCR4 and MMP-2 are overexpressed in pancreatic cancer tissues, and have been found to act as prognostic markers in various types of cancer, including pancreatic cancer. SDF-1/CXCR4 are thought to promote pancreatic cancer cell invasion by upregulating the expression and activity of MMP-2 [14–17]. Inhibition of GSK3β enhanced Mesenchymal stromal cells (MSCs) migration by increasing expression of CXCR4 [18]. SDF1/CXCR4 had effect on resident c-kit(+) CSPCs by increasing GSK3β activity [19]. Silencing of GSK3β decreased CXCL12 expression (the ligand of CXCR4) [20].

Here, we investigated the relationship between GSK3β and CXCR4/MMP-2 in pancreatic cancer cells, in order to further characterize the cellular and molecular mechanisms involved in pancreatic cancer.

## Results

### Overexpression of GSK3β upregulates CXCR4 and MMP-2

**Fig. 1:**
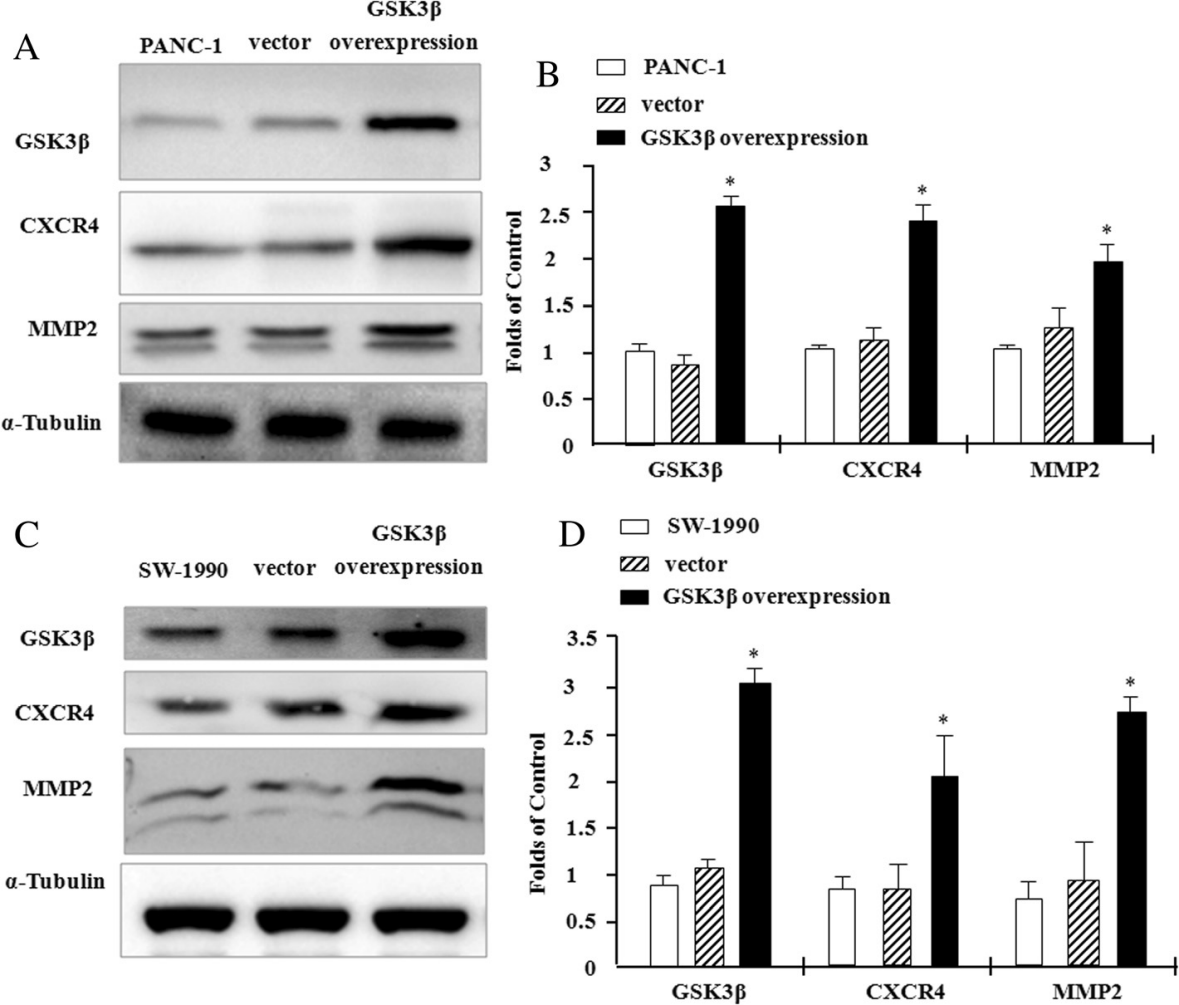
Overexpression of GSK3β upregulates CXCR4 and MMP-2 protein expression. PANC1 and SW-1990 human pancreatic cancer cells were transfected with either the GSK3β overexpressing plasmid pcDNA3.1A(-)-myc-GSK3β or empty vector as a control, and stable cell clones were selected. Total cell lysates were isolated and subjected to Western blotting using anti-human GSK3β, CXCR4 and MMP-2 antibodies; α-tubulin was used as a loading control. **a** Western blot analysis of GSK3β, CXCR4 and MMP-2 protein expression in control, vector control and GSK3β-overexpressing PANC1 cells. **c** Western blot analysis of GSK3β, CXCR4 and MMP-2 protein expression in control, vector control and GSK3β-overexpressing SW-1990 cells. **b** and **d** Quantification of GSK3β, CXCR4 and MMP2 expression in **a** and **c** based on gray-scale analysis. Results are expressed as mean ± SD; *p < 0.05 compared to control cells

To elucidate the effect of GSK3β on the expression of CXCR4 and MMP-2 in pancreatic cancer cells, we overexpresses GSK3β in PANC1 and SW-1990 cells. Fig. 1a show that, compared to control PANC1 cells and vector control cells, GSK3β expression increases by about 2.3-fold in the stable cell clone; this clone is used as a cellular model of GSK3β overexpression in the remainder of the study.

We determine the expression of CXCR4 and MMP-2 in PANC1 cells overexpressing GSK3β using Western blotting. Overexpression of GSK3β upregulates CXCR4 and MMP-2 expression by about 2.4- and 2.1-fold, respectively, compared to control PANC1 cells and vector control cells (Fig. 1b and Additional file 1: Figure S2A). And the same phenomenon was aslo observed in SW-1990 cells (Fig. 1c and d).

### Inhibition of GSK3β downregulates CXCR4 and MMP-2 expression

**Fig. 2:**
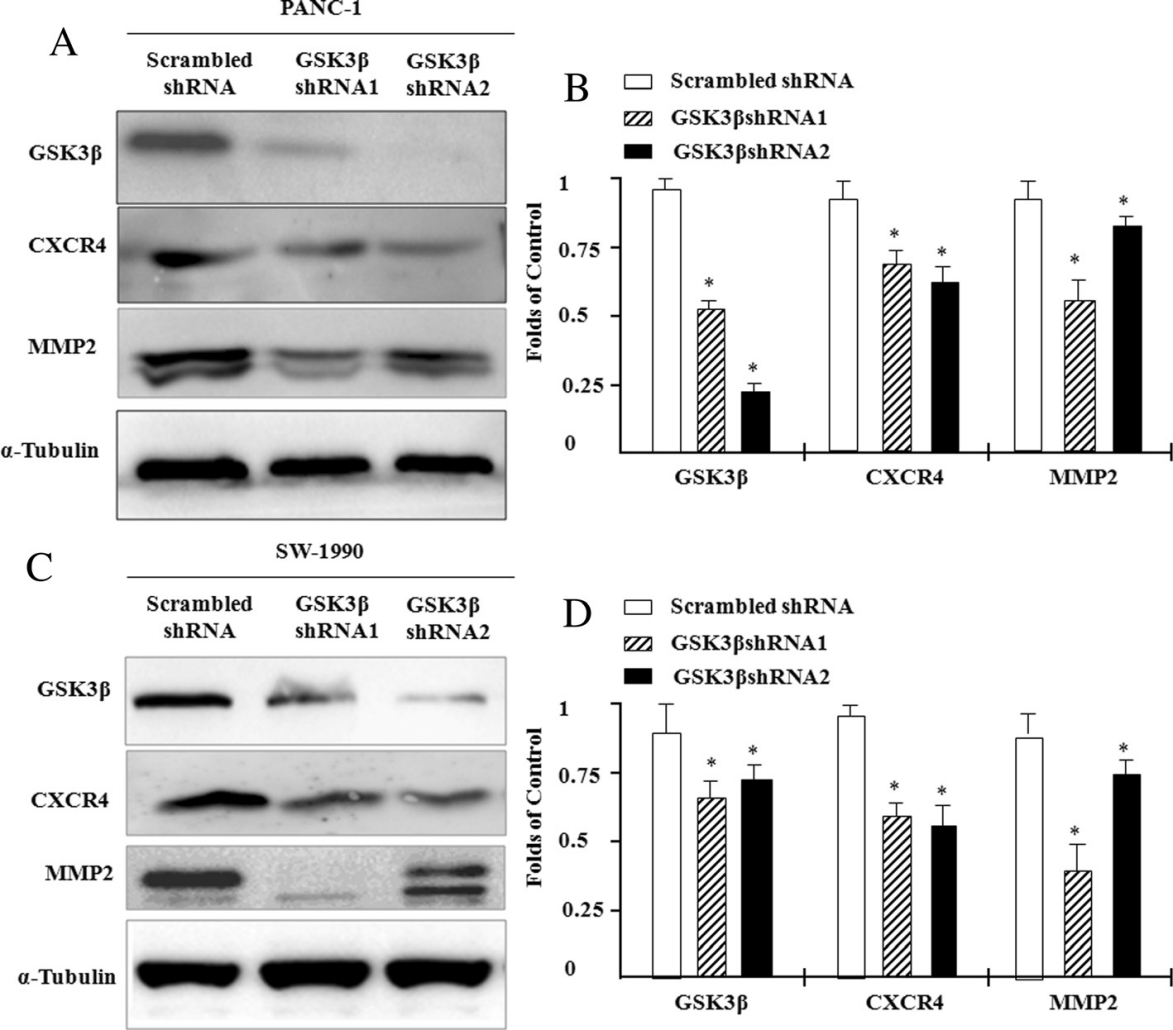
Inhibition of GSK3β downregulates CXCR4 and MMP2 expression. PANC1 and SW-1990 human pancreatic cancer cells were transfected with the GSK3β silencing plasmid pcDNA6.2-GW/EmGFP-miR-GSK3β expression vector, or scrambled vector as a control, and stable cell clones were selected. Total cell lysates were isolated and subjected to Western blotting using anti-human GSK3β, CXCR4 and MMP-2 antibodies; α-tubulin was used as a loading control. **a** GSK3β, CXCR4 and MMP-2 protein expression in PANC1 cells. (C) GSK3β, CXCR4 and MMP-2 protein expression in SW-1990 cells. **b** and **d** Quantification of GSK3β, CXCR4 and MMP-2 expression in **a** and **c** based on gray-scale analysis. Results are expressed as the mean ± SD; *p < 0.05 compared to control cells

Furthermore, we assesse whether inhibition of GSK3β affected CXCR4 and MMP-2 expression in PANC1 and SW-1990 cells. Using two different types of RNA interference, GSK3β expression is successfully down-regulated (Fig. 2a and b). Scrambled shRNA is used as a control. Suppression of GSK3β expression significantly reduces CXCR4 and MMP-2 expression approximately 2.3 folds and 5.8 folds in PANC1 cells, compared to scrambled control cells (Fig. 2a, b and Additional file 1: Figure S2B). And the same phenomenon was aslo observed in SW-1990 cells (Fig. 2c and d). We also detected the effect of GSK3β inhibition on the expression of CXCR4 and activity of downstream signaling molecular β-catenin using GSK3-specific inhibitor AR-A014418. Additional file 2: Figure S1 showed that inhibition of GSK3β downregulated CXCR4 expression and suppressed phosphorylation of β-catenin.

### GSK3β promotes PANC1 cell invasion

**Fig. 3:**
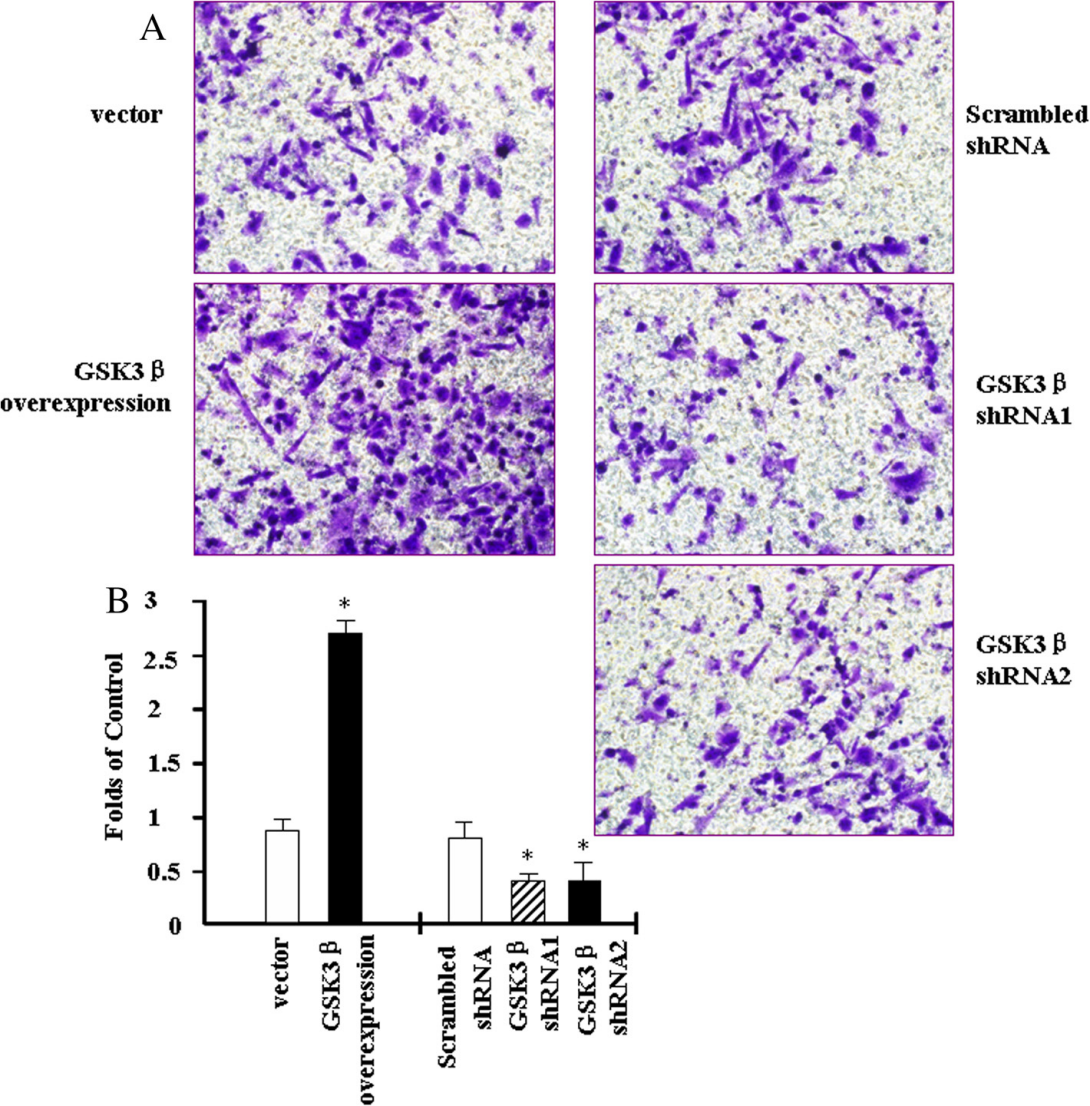
GSK3β promotes PANC1 human pancreatic cancer cells invasion. Transwell invasion assay was employed to determine the effect of GSK3β on cell invasion. Stable cell clones of PANC1 cells overexpression or suppression of GSK3β were selected. 200 μl cell suspension maitained in serum-free DMEM/0.1 % BSA were added to the matrigel-coated upper compartment, the DMED/10%FBS medium was added to the lower compartment, and the plates were incubated for 48 h at 37 °C. **a** After incubation for 48 h, the upper surface of the filter was scrubbed free of cells, the filter was fixed and stained and the lower surface was photographed (200× original magnification). **b** Quantification of A. Results are expressed as the mean ± SD; *p < 0.05 compared to control cells

We examine the effect of GSK3β on cell invasion in PANC1 cells using the *in vitro* cell invasion assay. As shown in Fig. 3a and b, the cell invasion assay demonstrates that overexpression of GSK3β promoted the invasion of PANC1 cells compared to vector control cells; suppression of GSK3β decreases the invasion of PANC1 cells compared to the scrambled cells. These results indicate that GSK3β induces PANC1 cell invasion.

### PANC1 cell invasion induced by overexpression of GSK3β is attenuated by suppression of CXCR4

**Fig. 4:**
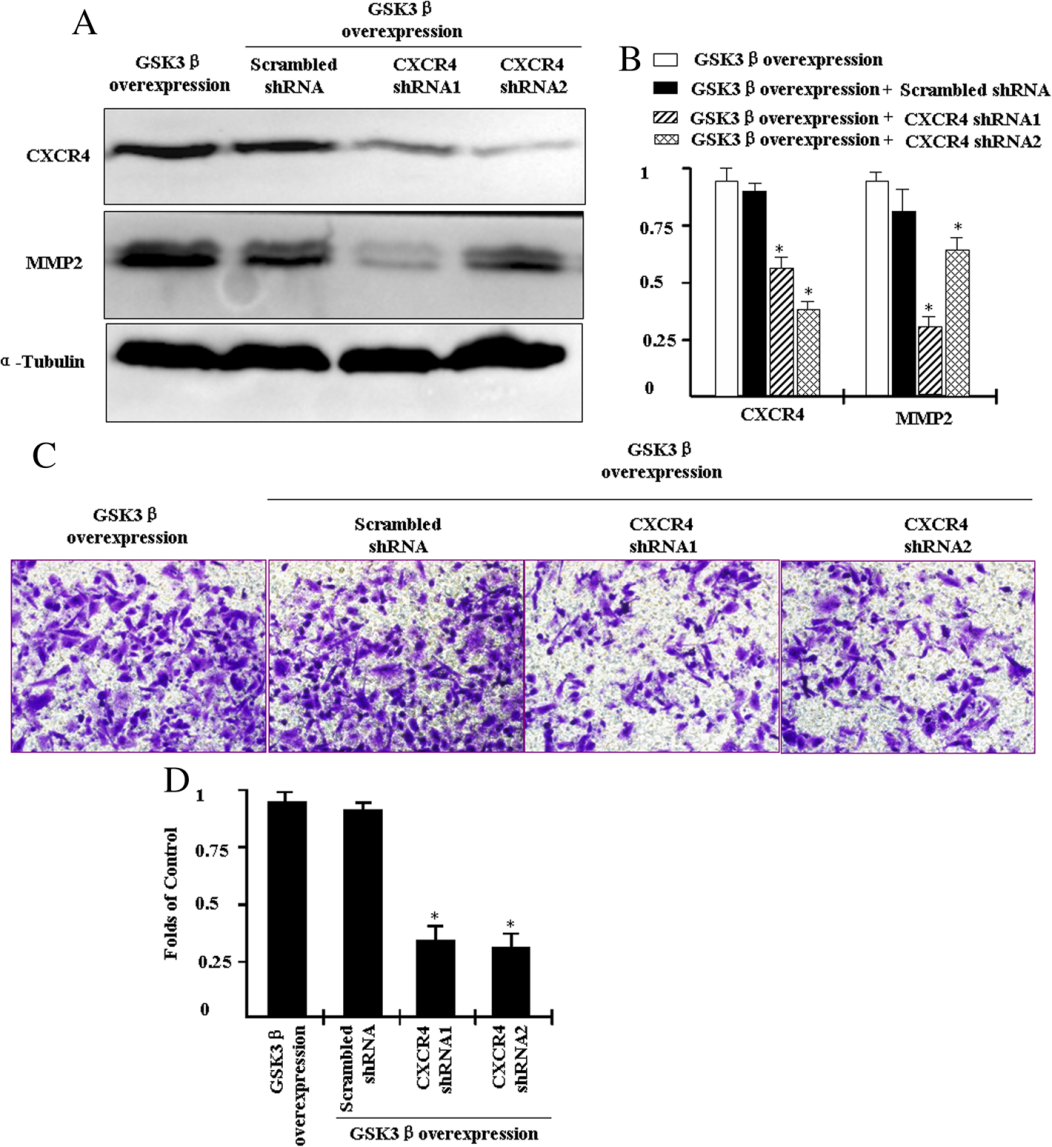
Overexpression of GSK3β promotes PANC1 cell invasion and MMP-2 expression via a CXCR4-dependent mechanism. To determine the effect of CXCR4 on cell invasion induced by GSK3β overexpression, PANC1 cells overexpression GSK3β were transfected with the CXCR4 silencing plasmids, or scrambled vector as a control, and stable cell clones were selected. **a** Total cell lysates were isolated and subjected to Western blotting using anti-MMP-2 and anti-CXCR4-antibodies; α-tubulin was used as a loading control. **b** Quantification of CXCR4 and MMP-2 expression in **a** based on gray-scale analysis. **c** Transwell invasion assay was employed to determine the effect of CXCR4 on PANC1 cells invasion induced by GSK3β overexpression. **d** Quantification of A. Results are expressed as the mean ± SD; *p < 0.05 compared to control cells

MMP-2 degrades the extracellular matrix, which promotes cancer cell invasion, and we previousl [21] found that CXCR4 promoted human cancer cell invasion by upregulating MMP-2. In this study, we observe that GSK3β regulated CXCR4 and MMP-2 expression (Figs. 1 and 2). Therefore, we investigated whether GSK3β regulated MMP-2 expression and cell invasion via CXCR4 signaling. To determine the effect of CXCR4 on cell invasion induced by GSK3β overexpression, PANC1 cells overexpression GSK3β were transfected with the CXCR4 silencing plasmids, or scrambled vector as a control, and stable cell clones were selected. Western blotting result showes that CXCR4 protein is successfully suppressed (Fig. 4a and b).

As shown in Fig. 4a and b, up-regulation of MMP-2 induced by overexpression of GSK3β is attenuated by the CXCR4 inhibition using transfecting with CXCR4 shRNA plasmids. Fig. 4c shows that overexpression of GSK3β also increased the invasion of PANC1 cells compared to the control cells. However, the ability of GSK3β to promote cell invasion is reduced by the CXCR4 inhibition. These results indicate that GSK3β induces MMP-2 expression and cell invasion via a CXCR4-dependent mechanism in PANC1 cells.

## Discussion

Among the current range of novel target molecules, GSK3β has emerged as a therapeutic target in pancreatic cancer [8, 22, 23]. GSK3β expression and activity are upregulated in pancreatic cancer [24–28]. In spite of this evidence, the precise role of GSK3β and its potential as a therapeutic target in pancreatic cancer still require further research. The focus of this study was to determine the effects of GSK3β and investigate its molecular mechanism of action, specifically via the GSK3β-CXCR4/MMP-2 pathway, in PANC1 pancreatic cancer cells.

Previous studies have shown that inhibition of GSK3β reduced the proliferation and survival of pancreatic cancer cells, which was associated with decreased cyclin D1 expression, Rb phosphorylation and secretion of matrix metalloproteinase-2 (MMP-2) [26, 29, 30]. Inhibition of GSK3β also suppressed pancreatic cancer growth and angiogenesis by decreasing the expression of Blc-2 and vascular endothelial growth factor (VEGF), and abrogating NFκB activity [17, 31]. GSK3β also maintained constitutive NFκB signaling in pancreatic cancer cells [9, 32–34]. The GSK3β/β-catenin pathway has also been linked to pancreatic cancer [35]. These results led us to propose GSK3β as a potential therapeutic target in pancreatic cancer; therefore, further studies on the molecular mechanism of action of GSK3β are required. The effect of GSK3β on SDF-1/CXCR4 is complicated. Kim YS et al. sugguested that inhibition of GSK3β upregulated expression of CXCR4 but Tamura M et al. said that silencing of GSK3β decreased SDF-1 expression [18, 20]. SDF-1 might increase GSK3β phosphorylation [19]. SDF-1/CXCR4 aslo mediated GSK3β-induced physiological migration of stem cells [36]. Activation of CXCR4-mediated cell signal resulted in the inhibition of GSK3β [37].

In our previous study [21], we found that SDF-1α/CXCR4 upregulated MMP-2 expression and induced the invasion of PANC1 and SW-1990 pancreatic cancer cells by activating p38 MAPK. Additionally, inhibition of GSK3β reduced the secretion of MMP-2 [26]. These results shed light on the molecular mechanism of action of GSK3β in pancreatic cancer. In this study, we demonstrated that GSK3β induced PANC1 pancreatic cancer cell invasion via the CXCR4/MMP-2 pathway.

## Conclusions

This study provides further insight into the molecular mechanism of GSK3β-induced pancreatic cancer invasion, and will allow exploration of novel therapeutic strategies for pancreatic cancer that target GSK3β and/or CXCR4/MMP-2.

## Materials and methods

### Reagents

Dulbecco’s modified Eagle’s medium (DMEM; #11965-084) was purchased from Life Technologies^TM^ (USA). The α-Tubulin antibody (#T5168) and AR-A014418 (#487021-52-3, an inhibitor of GSK3) were purchased from Sigma-Aldrich (USA). Antibodies against GSK3β (#9315) and MMP-2 (#4022) were purchased from Cell Signaling Technology (USA). Antibodies against CXCR4 (#ab2074) was purchased from Abcam.

### Cell culture

The human pancreatic cancer cell line PANC1 (CRL-1469) and SW-1990 (CRL-2172), which was established from human pancreatic cancer ducts, was purchased from the American Type Culture Collection (ATCC, Manassas, VA, USA). PANC1 cells were cultured in Dulbecco’s modified Eagle’s medium (DMEM) supplemented with 10 % fetal bovine serum, 100 U/ml penicillin and 100 mg/ml streptomycin sulfate, and maintained at 37 °C in a 5 % CO_2_ humidified incubator.

### Construction of GSK3β overexpressing plasmid and generation of stable clones

The overexpressing plasmid pcDNA3.1A(-)-myc-GSK3β was constructed as follows: full-length human *GSK3β* cDNA was amplified by RT-PCR from mRNA isolated from human white adipose tissue. The primer sequences were: forward, 5'-CGTGAATTCATCATGTCAGGGCGGCCCA -3' and reverse, 5'-GCTGTCGACG GGATCCGTCAGGTGGAGTTGGA-3'. The PCR product was cloned into the expression vector pcDNA3.1A(-)-myc, and then transfected into PANC1 cells using X-tremeGENE HP DNA Transfection Reagent. At 48 h after transfection, the cells were cultured in selection medium containing 800 μg/ml G418 (Sigma) to select resistant colonies for further analysis.

### Construction of miRNA-GSK3β expression plasmids and stable clone selection

Two distinct domains within the coding region of the human GSK3β cDNA were targeted for RNA interference. For this purpose, two pairs of reverse complementary oligonucleotides were designed and synthesized as Table 1. The oligonucleotides were annealed and inserted into the pcDNA6.2-GW/EmGFP-miR expression vector (Invitrogen, #K4936-00) to create GSK3β shRNA1 and shRNA2. A control scrambled shRNA was also created. We used X-tremeGENE HP DNA Transfection Reagent to separately transfect the three kinds of plasmids into PANC-1. At 48 h after transfection, the cells were cultured in selection medium containing 800 μg/ml G418 (Sigma) to select resistant colonies for further analysis. CXCR4 shRNA plasmids are gifts from the Department of Gastroenterology, The First Affiliated Hospital of Nanjing Medical University [38].

**Table 1:** Reverse complementary oligonucleotides

Oligo	5’ to 3
1-F	TACATAGTAAGTGGGCCTTCAGGCGCCTTTTGCGGTTTGACTGATGCTCTGAACTGCCGGCCTA
1-R	AGTGTAGTACTAGAGAACAGGCTAGGGAGTTTGGCCCCAACCACTGCCTGTTCTGTCCTTGCTC
2-F	GAAGAGGCAGGGATCAGTAGCGTAGGGTTTAAAACACTGACTGCTGCCTAGTTACTCCACTGAA
2-R	GCCAACAGTGTTGGAGCTAGGGACCGGTCAAACAGGCCAAAGCCTCCTAGCTACTTCCGGTTCC
Scrambled-F	TGTCTGAACATACTGCCTGAGAGACGTTCTGACCACTAAGAACGTCTCTGAACAGTACTGAT
Scrambled-R	TACTAAAGTCAACTGCGAAGGAGAAGTCAGTTGAGGCCCAGAACGTAGCCACGCTTACTACTA

### Western blotting analysis

PANC1 cells were lysed using RIPA buffer. Equal amounts of protein (60 μg) were separated on 10 % SDS-PAGE gels and transferred to Immobilon-P membranes (Millipore, Bedford, MA, USA). The membranes were probed overnight at 4 °C with antibodies against GSK3β, CXCR4 or MMP-2 in TBST containing 1 % (*w/v*) BSA, incubated with the appropriate anti-rabbit or anti-mouse secondary antibodies for 2 h, and immune complexes were detected using the ECL plus detection kit (Pierce, Rockford, IL, USA) and quantified using a scanning densitometer and molecular analysis software (Bio-Rad, Hercules, CA, USA).

### Cell invasion assay

PANC1 cell invasion was assayed using 24-well Transwell plates (Corning Costar, Schiphol-Rijk, Netherlands). ECM gel solution (60 μl) was added to the top compartment of each cell culture insert and dried overnight in a laminar flow cabinet. PANC1 cells were washed twice with phosphate-buffered saline (PBS), resuspended in serum-free DMEM containing 0.1 % BSA, and adjusted to a final concentration of 1 × 10^6^ cells/ml. Serum-free DMEM/0.1 % BSA (600 μl) and 200 μl cell suspension were added to the matrigel-coated upper compartment, and the same medium was added to the lower compartment, and the plates were incubated for 48 h at 37 °C. Cells remaining on the upper side of the filter were removed by gentle wiping, and cells that had migrated through the filter were counted using a light microscope (six fields were counted in each chamber for each condition; 200× magnification).

### Statistical analysis

Data were analyzed by two-tailed Student *t* test for single comparisons and by one-way analysis of variance for multiple group comparisons. Differences were considered significant at *p < 0.05 versus control.

## Additional files

Additional file 1: Figure S2. The transcript levels for CXCR4 and MMP2 were quantified by real-time quantitative transcription polymerase chain reaction (real-time PCR). Total RNA of PANC-1 cells were extracted by TRIzol reagent (Invitrogen) and 1 μg total RNA was used for reverse transcription to cDNA according to the manufacturer’s instructions. The cDNA aliquots were used for quantification of mRNA by real-time PCR using ABI Prism 7000 sequence detection system (Applied Biosystems, Foster City, CA), using SYBR Green PCR master mix (Applied Biosystems). All data were analyzed using β-actin gene expression as an internal standard. The specific primers were as follows: β-actin forward: 5′-TCACCAACTGGGACGACAT-3′, and reverse: 5′-GCACAGCCTGGATAGCAAC-3′, CXCR4 (NM_001008540.1) forward: 5′-CGAGGCCCTAGCTTTCTTCC-3′, and reverse: 5′-GAGGATCTTGAGGCT GGACC-3′, MMP2 (NM_001302509.1) forward: 5′-GGATGGCAAGTACGGCTTCT-3′, and reverse: 5′-GTT CCCACCAACAGTGGACA-3′. The thermal cycler was programmed as: denaturation at 95 °C for 5 min, followed by 33 cycles at 95 °C for 30 s and annealing at 60 °C for β-actin, 60 °C for CXCR4, and 56 °C for MMP2 for 30 s, extension in all was carried at 72 °C for 1 min, with a final extension step of 10 min. (A) Overexpression of GSK3β upregulates CXCR4 and MMP-2 expression respectively, compared to control PANC1 cells and vector control cells. (B) Suppression of GSK3β expression significantly reduces CXCR4 and MMP-2 expression approximately 2.3 folds and 5.8 folds in PANC1 cells, compared to scrambled control cells. Additional file 2: Figure S1. Effect of GSK3 inhibition on phosporylation of β-catenin and CXCR4 expression. The levels of phosphorylation of β-catenin and CXCR4 expression were examined by Western blotting in pancreatic cancer cells PANC-1 after treatment with 10 μM AR-A014418 (AR) (#487021-52-3, an inhibitor of GSK3, was purchased from Sigma-Aldrich (USA)) for 6 hous. Expression of CXCR4 and β-catenin and its phosphorylation (p-β-catenin S33/37/T41) (Phospho-β-catenin (Ser33/37/Thr41) #9561 antibody and β-catenin antibody #9562 were purchased from CST) were examined and compared between the same pancreatic cancer cells treated with DMSO or AR-A014418.

## Abbreviations

GSK3β: Glycogen synthase kinase-3β
CXCR4: Chemokine receptor C-X-C motif chemokine receptor 4
MMP-2: Matrix metalloproteinase 2
SDF-1: Stromal cell-derived factor-1
