# Supplementary figures and images for: GSK3β mediates pancreatic cancer cell invasion in vitro via the CXCR4/MMP-2 Pathway
Source: Cancer Cell Int. 2015 Jul 5;15:70. doi: 10.1186/s12935-015-0216-y (PMC4513390; doi:10.1186/s12935-015-0216-y)

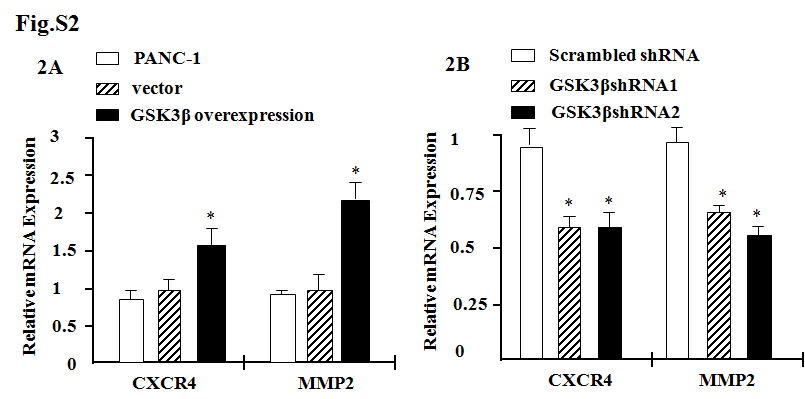

Supplement: Additional file 1: Figure S2. — The transcript levels for CXCR4 and MMP2 were quantified by real-time quantitative transcription polymerase chain reaction (real-time PCR). Total RNA of PANC-1 cells were extracted by TRIzol reagent (Invitrogen) and 1 μg total RNA was used for reverse transcription to cDNA according to the manufacturer’s instructions. The cDNA aliquots were used for quantification of mRNA by real-time PCR using ABI Prism 7000 sequence detection system (Applied Biosystems, Foster City, CA), using SYBR Green PCR master mix (Applied Biosystems). All data were analyzed using β-actin gene expression as an internal standard. The specific primers were as follows: β-actin forward: 5′-TCACCAACTGGGACGACAT-3′, and reverse: 5′-GCACAGCCTGGATAGCAAC-3′, CXCR4 (NM_001008540.1) forward: 5′-CGAGGCCCTAGCTTTCTTCC-3′, and reverse: 5′-GAGGATCTTGAGGCT GGACC-3′, MMP2 (NM_001302509.1) forward: 5′-GGATGGCAAGTACGGCTTCT-3′, and reverse: 5′-GTT CCCACCAACAGTGGACA-3′. The thermal cycler was programmed as: denaturation at 95 °C for 5 min, followed by 33 cycles at 95 °C for 30 s and annealing at 60 °C for β-actin, 60 °C for CXCR4, and 56 °C for MMP2 for 30 s, extension in all was carried at 72 °C for 1 min, with a final extension step of 10 min. (A) Overexpression of GSK3β upregulates CXCR4 and MMP-2 expression respectively, compared to control PANC1 cells and vector control cells. (B) Suppression of GSK3β expression significantly reduces CXCR4 and MMP-2 expression approximately 2.3 folds and 5.8 folds in PANC1 cells, compared to scrambled control cells. [file 12935_2015_216_MOESM1_ESM.tiff]

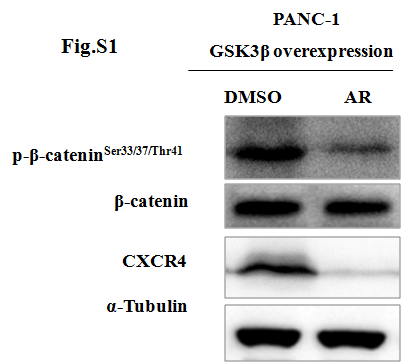

Supplement: Additional file 2: Figure S1. — Effect of GSK3 inhibition on phosporylation of β-catenin and CXCR4 expression. The levels of phosphorylation of β-catenin and CXCR4 expression were examined by Western blotting in pancreatic cancer cells PANC-1 after treatment with 10 μM AR-A014418 (AR) (#487021-52-3, an inhibitor of GSK3, was purchased from Sigma-Aldrich (USA)) for 6 hous. Expression of CXCR4 and β-catenin and its phosphorylation (p-β-catenin S33/37/T41) (Phospho-β-catenin (Ser33/37/Thr41) #9561 antibody and β-catenin antibody #9562 were purchased from CST) were examined and compared between the same pancreatic cancer cells treated with DMSO or AR-A014418. [file 12935_2015_216_MOESM2_ESM.tiff]
